# Supplementary material for: Causal interplay between lactose intolerance and gut microbiota: a combined bidirectional Mendelian randomization and in vivo validation study
Source: Front Nutr. 2026 Jun 1;13:1803337. doi: 10.3389/fnut.2026.1803337 (PMC13265576; doi:10.3389/fnut.2026.1803337)
Supplement: Supplementary file 1 [file Data_Sheet_1.zip › supplementary materials/Forward/leave one out cross validation/ebi-a-GCST90027664.finngen_R12_E4_LACTONAS.pdf]

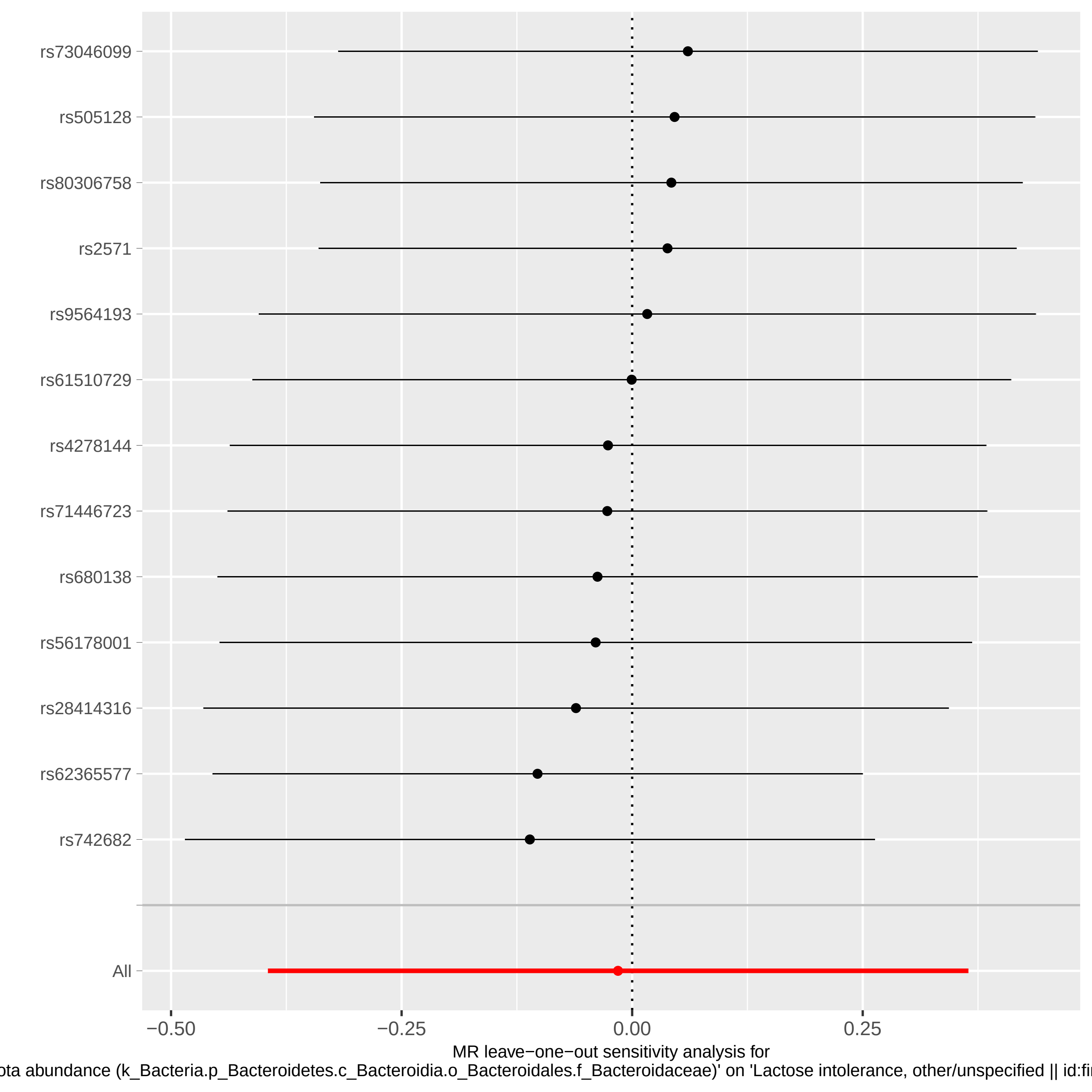
rs73046099
rs505128
rs80306758
rs2571
rs9564193
rs61510729
rs4278144
rs71446723
rs680138
rs56178001
rs28414316
rs62365577
rs742682
All
−0.50
−0.25
0.00
0.25
MR leave−one−out sensitivity analysis for
ota abundance (k_Bacteria.p_Bacteroidetes.c_Bacteroidia.o_Bacteroidales.f_Bacteroidaceae)' on 'Lactose intolerance, other/unspecified || id:fi
